# Health-Promoting Effects of Bioactive Compounds from Plant Endophytic Fungi

**DOI:** 10.3390/jof9100997

**Published:** 2023-10-08

**Authors:** Tharuka Wijesekara, Baojun Xu

**Affiliations:** 1 Department of Food Science and Technology, University of Peradeniya, Peradeniya 20400, Sri Lanka; minoliwijesekara777@gmail.com; 2 Food Science and Technology Program, Department of Life Sciences, BNU-HKBU United International College, Zhuhai 519087, China

**Keywords:** plant endophytic fungi, bioactive compounds, human health, mechanism of action, pharmaceuticals

## Abstract

The study examines the intricate relationship between plants and the endophytic fungi inhabiting their tissues. These fungi harmoniously coexist with plants, forming a distinct symbiotic connection that has caught scientific attention due to its potential implications for plant health and growth. The diverse range of bioactive compounds produced by these fungi holds significant promise for human health. The review covers various aspects of this topic, starting by introducing endophytic microorganisms, explaining their colonization of different plant parts, and illuminating their potential roles in enhancing plant defense against diseases and promoting growth. The review emphasizes the widespread occurrence and diversity of these microorganisms among plant species while highlighting the complexities and significance of isolating and extracting bioactive compounds from them. It focuses on the health benefits of these bioactive compounds, including their capacity to exhibit antioxidant, anti-inflammatory, antimicrobial, and anticancer effects. The review delves into the mechanisms behind these health-promoting effects, spotlighting how the compounds interact with cellular receptors, signaling pathways, and gene expression. In conclusion, the review provides a comprehensive overview of health-promoting bioactive compounds from plant endophytic fungi. It outlines their multifaceted impact, potential applications, and future research avenues in health and medicine.

## 1. Introduction

Endophytic microorganisms are commonly found on plants, especially perennial varieties. These microorganisms, which include both fungi and bacteria, occupy plant tissues for prolonged periods or specific phases of their life cycles [1]. Importantly, this colonization takes place without causing discernible harm or inducing visible changes in the physical appearance of the host plants. This intricate association has garnered attention due to its potential implications for plant well-being and development [2]. Endophytes are harmless microorganisms that live inside healthy plant tissues and protect them from diseases. They achieve this through strategies such as colonization, competition for nutrients, antibiotic synthesis, and resistance mechanisms [3]. Endophytic fungi not only safeguard plants but also enhance growth by producing phytohormones, bolstering stress resistance, and creating protective pesticides [4]. Due to their capacity to produce a variety of bioactive substances with a significant potential for enhancing human health, these mysterious microorganisms have carved out an intriguing habitat within the living parts of plants [2]. Scientists have found evidence of at least one type of endophyte in every plant examined so far [5]. These endophytes can take up residence in different parts of plants, including the stem, roots, leaves, inflorescences, fruits, seeds, and even in non-living parts such as dead plants [6]. The number of endophytes in a particular plant species can vary significantly and depends on factors such as the type of plant, its stage of growth, the density of the invading microorganisms, and the surrounding environmental conditions [7]. These fungi are ubiquitous and have the potential to produce a wide variety of bioactive molecules due to their inherent diversity, which includes a wide range of plant species. This review will then continue to explore the challenging procedures for obtaining and isolating these priceless bioactive compounds from endophytic fungi [8]. This is a difficult task that requires understanding the intricate habitats of these fungi within plant tissues and developing strategies to maximize the inherent potential of these compounds [9].

Transitioning to the heart of this exploration, an in-depth analysis of the health-promoting properties exhibited by these bioactive compounds is embarked upon. Their potential as antioxidants, which are capable of neutralizing harmful free radicals and safeguarding cellular health, forms a critical cornerstone of their benefits [10]. At the same time, their anti-inflammatory properties hold promise for reducing chronic inflammatory conditions that are behind many health problems. The compounds’ effectiveness as antimicrobial agents makes a significant contribution to the ongoing battle against drug-resistant pathogens, which further highlights their importance [11]. Moreover, their potential to combat cancer and modulate immune responses adds layers of complexity to their potential applications in health promotion [12]. This review offers an overview of the molecular pathways responsible for these effects by providing insights into the complex interactions that guide the actions of these compounds. By investigating how these compounds interact with cellular targets and affect gene expression, the intricate network of interactions that supports their potential for improving health is uncovered. Transitioning from theoretical potential to real-world impact, this exploration delves into in vivo studies and clinical trials [13]. Delving into animal studies assessing the health benefits of these bioactive compounds provides insights into their impact on overall well-being. Human clinical trials, serving as a bridge between laboratory promise and real-world application, offer a nuanced understanding of the effects of these compounds on human health [14]. In the pursuit of therapeutic advancement, safety and toxicity considerations emerge as essential factors to be addressed. The practical implications of these bioactive compounds come to the forefront with the exploration of their potential applications in health and medicine [15]. Nutraceuticals and functional foods have the potential to reshape dietary habits; pharmaceutical drugs may revolutionize medical treatments; and the integration of these compounds into health supplements offers prospects that are both diverse and promising [16]. Identifying and isolating novel bioactive compounds remain key challenges that demand innovative approaches. Ensuring standardization and quality control of endophytic-derived products is essential for consistency and safety. Understanding the intricate interactions between these bioactive compounds and the human body is a puzzle that requires ongoing investigation [17].

In the concluding stages of the exploration, the review summarizes the health-promoting effects of bioactive compounds from plant endophytic fungi, emphasizing their potential contributions across a spectrum of health areas. Outlining directions for future research and application in health and medicine points to a future where treasures are hidden within these fungi. Unveiling the health-promoting potential of bioactive compounds from plant endophytic fungi involves embarking on a journey that merges the realms of nature and science, paving the way for a healthier future.

## 2. Plant Endophytic Fungi and Their Bioactive Compounds

### 2.1. Definition and Characteristics of Endophytic Fungi

Plant endophytic fungi reside within the living tissues of plants, forming a unique symbiotic relationship that has captured the attention of researchers [18]. Endophytic fungi are organisms that inhabit the internal tissues of plants without causing any apparent harm or disease symptoms to their host. Unlike parasites or pathogens, they form a mutualistic association with the plant, from which both parties benefit [19]. This intricate relationship often involves the exchange of nutrients, signals, and metabolites, contributing to the overall health and resilience of the plant. These fungi possess distinct characteristics that set them apart from other microbial inhabitants [20]. One of their defining features is their ability to exist within the plant for extended periods, often throughout the plant’s life cycle. They colonize various plant tissues, including leaves, stems, and roots, and display remarkable adaptability to different environmental conditions [21]. Unlike pathogens, they do not provoke an immune response from the host plant, allowing them to maintain a stealthy coexistence. Endophytic fungi have evolved diverse mechanisms to survive within the plant’s internal environment [22]. Some form specialized structures called “microsclerotia” or “sclerotia”, which protect them from adverse conditions. Others produce secondary metabolites, including bioactive compounds, which contribute to their ecological success and their potential to influence plant health [23]. This article unravels the myriad ways in which these endophytic fungi and their bioactive compounds contribute to the promotion of human health. The journey ahead involves uncovering their potential as sources of antioxidants, anti-inflammatory agents, antimicrobial compounds, and more, highlighting the profound impact they may have on human well-being.

### 2.2. Diversity of Endophytic Fungi in Different Plant Species

Endophytic fungi have established themselves as versatile inhabitants by finding their niche within the internal tissues of plants across various ecosystems [24]. This section explores the remarkable diversity of endophytic fungi and their distribution within different plant species [25]. They are not found in just one type of plant. They live in many different kinds of plants such as trees, bushes, grass, and small plants. These fungi can live in many different places such as forests, grassy areas, wetlands, and even underwater. This remarkable adaptability has led to their presence in diverse habitats, including forests, grasslands, wetlands, and even aquatic ecosystems [26]. Endophytic fungi exhibit a high degree of phylogenetic diversity and have been discovered in all terrestrial plants investigated to date. Among the most commonly isolated genera are Penicillium, Alternaria, Fusarium, Colletotrichum, Aspergillus, and Xylaria [27]. However, other studies have also identified genera such as Alternaria, Colletotrichum, Fusarium, Gibberella, Glomerella, Guignardia, Leptosphaerulina, Nigrospora, Phoma, Phomopsis, and Xylaria. It is important to note that the frequency of isolation of these genera can vary depending on factors related to the host plant, including its genotype, the specific tissue sampled, geographical location, plant age, and the season in which the sampling is conducted [28]. The distribution of endophytic fungi is not only influenced by the plant’s taxonomy but also by its geographical location and environmental conditions. Different plant species harbor distinct endophytic communities, reflecting the intricate interplay between the fungi and their host plants [29]. Moreover, the surrounding soil, climate, and even the presence of neighboring plants can influence the composition and diversity of endophytic fungi within a given plant species. The diverse array of endophytic fungi is not merely a result of their passive colonization. It is a testament to the complex and dynamic relationship they share with their host plants [30]. The diverse functions that these fungi fulfill contribute to their distribution patterns. Some endophytic fungi assist their host plants by enhancing nutrient uptake, improving water efficiency, and mitigating stressors such as drought and pathogens [31]. In turn, the host plants provide a sheltered environment and access to essential nutrients, fostering a symbiotic exchange that fuels their coexistence [32]. The distribution and diversity of endophytic fungi also provide a glimpse into their potential applications. Different plant species may serve as reservoirs for unique bioactive compounds, offering a wellspring of possibilities for harnessing their health-promoting effects [33]. The diversity of endophytic fungi will emerge as a cornerstone, underscoring their significance in reshaping our understanding of wellness and healthcare paradigms.

### 2.3. Extraction and Identification of Bioactive Compounds from Endophytic Fungi

Endophytic fungi have emerged as a treasure trove of bioactive compounds that could revolutionize health and medicine. These fungi synthesize an array of molecules with potential health-promoting effects [34] that could pave the way for innovative healthcare interventions [15]. Utilizing a variety of extraction techniques, each one is tailored to the unique properties of the bioactive substances and the under-researched fungal strains [35]. Solvent-based methods, such as maceration and Soxhlet extraction, are commonly used to dissolve the compounds in organic solvents. Supercritical fluid extraction and microwave-assisted extraction offer alternatives that leverage the unique properties of the solvents used [36]. For extraction from culture liquid, ethyl acetate is the best solvent [37]. After the extraction process, the subsequent step involves isolating the bioactive compounds from the complex mixture obtained from endophytic fungi. Chromatographic techniques, including methods such as thin-layer chromatography (TLC) and high-performance liquid chromatography (HPLC), play a crucial role in separating this mixture into individual compounds based on their distinct physicochemical properties [38].

The isolated compounds are subjected to rigorous characterization and identification processes to elucidate their chemical structures and properties. Techniques such as nuclear magnetic resonance (NMR) spectroscopy and mass spectrometry (MS) play a crucial role in unraveling the molecular structures of these compounds [39]. This phase is essential to validate the presence of bioactive compounds and understand their potential health-promoting properties. The journey from extraction to isolation is a testament to the complexity and potential of endophytic fungi and their bioactive compounds.

### 2.4. Bioactive Compounds of Endophytic Fungi

Endophytic fungi have emerged as a hopeful reservoir of innovative and environmentally friendly bioactive compounds, exhibiting minimal toxicity. Phytochemicals are classified based on their structural characteristics among bioactive metabolites. They encompass a wide range of biological properties, including antimicrobial, immunosuppressive, antiparasitic, antioxidant, anti-inflammatory, and anticancer effects, among others [40]. They surpass conventional antimicrobials in terms of efficacy, cost-effectiveness, and reduced susceptibility to microbial resistance. This presents a promising solution to the substantial challenges confronting public health systems. Moreover, from an economic perspective, the industrial utilization of fungal endophytes as a source of bioactive compounds holds significant potential for substantial benefits within the medical sector [10]. Metabolites derived from fungal endophytes, renowned for their antimicrobial properties, encompass a range of compounds, including altersolanol A, clavatol, chaetomugilin D, colletotric acid, enfumafungin, guignardic acid, hydroxy-jesterone, jesterone, pestacin, rutin, viridicatol, 2-hydroxyl-6-methyl benzoic acid, 7-amino-4-methylcoumarin, and xylarenone B, among others [2]. An exceptional endophyte found in Citrus nobilis, specifically Streptomyces spp. TQR12–4, has been documented to possess broad-spectrum antifungal capabilities, effectively targeting fungal pathogens such as *Colletotrichum* spp., *Geotrichum* spp., *Fungus* spp., and others [41]. Endophytes such as *Juniperus communis*, *Phialocephala fortini*, and *Trametes hirsute*, inhabiting the tissues of *Juniperus recurve* and *Podophyllum peltatum*, produce podophyllotoxin, a well-known biomolecule recognized for its anticancer attributes [42]. Extensive research has unveiled the diverse bioactivities exhibited by metabolites produced by various fungal endophytes. For instance, *Chaetomium* spp. and *Xylaria* spp., which are associated with the herbal plants *Nerium oleander* and *Ginkgo biloba*, have been identified as potent antioxidants [43]. Endophytes have demonstrated their potential for possessing antidiabetic properties. For instance, *Acacia nilotica* is known to host the endophyte *Aspergillus awamori*, which has been scientifically verified to produce bioactive compounds with efficacy against diabetes [44]. Certain metabolites produced by endophytic fungi have been documented for their effectiveness against various viruses, including the dengue virus, HIV, human cytomegalovirus, and influenza A (H1N1) virus. Additionally, two novel compounds, namely cytonic acid A (C_32_H_36_O_10_) and cytonic acid B (C_32_H_36_O_10_), have been synthesized by *Cytonaema* spp. and are reported to possess antiviral properties [40]. Subglutinol A and colutellin A have repeatedly demonstrated their potential as effective immunosuppressants for the treatment of various immunological disorders. These compounds have been extracted from fungi residing within plant tissues [45].

### 2.5. Plant and Endophytic Fungal Interactions

The connection between endophytic fungi and plants has evolved gradually, with specific adaptations from both the plant and the fungi. This relationship is intricate and varies depending on the specific fungus, plant species, or a combination of both [46]. There are two prevailing theories explaining the source of endophytes: Exogenous and endogenous. According to the endogenous theory, endophytes originate from plant chloroplasts and mitochondria, sharing genetic histories with their host plants. Conversely, the exogenous hypothesis proposes that endophytes enter their host plants through various means such as surface contact, induced channels, or root wounds [47]. Over time, endophytic fungi and their host plants have developed a range of associations, spanning from (i) mutualistic relationships to (ii) antagonistic ones, and even (iii) neutral interactions. Some fungal endophytes remain mostly inactive within their host tissues throughout the plant’s life, a state referred to as neutralism. Others may remain dormant until environmental conditions become favorable, leading to either mutualistic or antagonistic interactions [48]. Endophytic fungi have evolved three distinctive modes of reproduction: Firstly, there is vertical transmission, also known as intercellular transmission, where infected plants pass on the fungal infection to their offspring through seeds or hyphae. An exemplar of this transmission method is *Neotyphodium* sp. Secondly, horizontal transmission, which is intracellular in nature, takes place when sexual spores from infected plants disperse to initiate infections, and this mechanism is employed by *Epichloe* spp. [49]. Lastly, there is a mixture transmission approach, which combines both vertical and horizontal transmission strategies. Vertical transmission is an asexual means of reproduction involving the transfer of intercellular hyphae from infected plants to non-plant hosts, initiating infection or germinating infected seeds. In contrast, horizontal transmission involves the development of infectious sexual spores that undergo the fungal sexual life cycle [40].

## 3. Health-Promoting Properties of Bioactive Compounds of Plants Endophytic Fungi

### 3.1. Antioxidant Activity

Oxidative stress is a condition defined by an uneven balance between the production of free radicals and the body’s capacity to counteract them, and it plays a crucial role in the initiation and advancement of multiple diseases. These free radicals, which are unstable molecules possessing unpaired electrons, cause significant disruption within cells, ultimately resulting in cellular damage, inflammation, and the hastening of aging-related processes [50]. The effects of oxidative stress are widespread, affecting everything from cardiovascular diseases to neurodegenerative disorders. The realm of plant endophytic fungi contains a variety of bioactive substances with strong antioxidant properties [51]. Polyphenols, flavonoids, terpenoids, and various phenolic compounds are among the remarkable array of antioxidant molecules synthesized by these fungi. These compounds possess the remarkable capacity to neutralize free radicals, serving as potent defenders against oxidative damage that underscores diverse health issues [52]. These bioactive compounds’ antioxidant properties go beyond simply squelching free radicals. They have the potential to serve as a protective shield, bolstering cellular integrity against the onslaught of oxidative stress. These substances aid in the reduction of inflammation, the prevention of chronic diseases, and the preservation of cellular processes vital to longevity and good health by limiting the cellular damage brought on by free radicals [53]. The effects of antioxidants found in endophytic compounds have important consequences for human well-being. They provide a proactive method to prevent diseases by strengthening the body’s natural defenses against harmful oxidative processes. These benefits also apply to skin health, where these compounds can be used to combat premature aging and enhance the skin’s resilience to external aggressors [54]. Recent investigations have affirmed that *Aspergillus* represents the prevailing fungal endophytes known for generating antioxidants. A study underscored that *A. flavus*, *A. fumigatus*, and *A. nidulans* exhibit noteworthy antioxidant potential, with IC_50_ values spanning 68.4–347.1 µg/mL [15]. In a similar study, it was found that *Aspergillus minisclerotigens* AKF1 and *Aspergillus oryzae* DK7 isolated from *Mangifera casturi* Kosterm and that both of these fungi displayed antioxidant efficacy, with IC_50_ values of 142.96 and 145.01 µg/mL, respectively [55]. The extract obtained from endophytic *A. nidulans*, sourced from *Passiflora incarnata*, exhibited substantial antioxidant potential [56]. A study indicates that the endophytic fungus isolated from the *M. luteola* plant exhibited a wide array of biological activities and contained phytochemicals. The ethyl acetate extract of *Alternaria* sp. (ML4) demonstrated an 85.20% inhibition activity against DPPH, which is nearly comparable to the inhibition activity of ascorbic acid at 96.91%. Additionally, this extract exhibited high reducing power activity in comparison to standard ascorbic acid. Moreover, it possessed the highest total phenolic and flavonoid concentrations, measuring 108.65 ± 0.12 mg of GAE/g and 56.45 ± 0.10 mg of RE/g of extract, respectively [57]. These results set the stage for investigating how these compounds could be used in human health, which could potentially lead to new approaches in preventive medicine.

### 3.2. Anti-Inflammatory Effects

While inflammation functions as the body’s defense mechanism, it can occasionally lead to issues. It can harm our tissues and cause chronic diseases. Chronic inflammation is a risk factor for diseases such as heart disease, autoimmune disorders, and some cancers. Therefore, reducing the negative effects of inflammation is crucial for maintaining our general health [58]. The bioactive compounds of endophytic fungi are known for their anti-inflammatory properties. These substances, which range from polyphenols to terpenoids, have shown that they can alter inflammatory pathways and lessen the series of actions that lead to chronic inflammation. They have the potential to reduce the risk of several diseases caused by inflammation through these actions [59]. The anti-inflammatory properties of endophytic compounds go beyond merely reducing inflammation; they also have the power to balance cellular reactions. These substances enable a well-balanced immune reaction by reducing the production of pro-inflammatory molecules. This fine-tuned regulation affects the body’s capacity to handle stress and maintain homeostasis beyond disease prevention [60]. These anti-inflammatory effects have broad-ranging implications for health and well-being. These substances provide a comprehensive approach to addressing not only disease prevention but also the management of existing conditions by dousing the flames of chronic inflammation. The current study found that *P. brefeldianum*, an endophytic fungus isolated from *A. hispida* leaves, had an effective anti-inflammatory action in the carrageenan-induced paw edema model at a concentration of 200 mg/kg. The modified histological and immunohistochemical features of the paw skin sections, as well as the decrease in inflammatory and oxidative stress biomarkers, have been revealed by ELISA and qRT-PCR [61]. Animal studies showcasing the reduction of inflammatory markers and the mitigation of tissue damage lay the foundation for future applications in human health.

### 3.3. Antimicrobial Properties

The conflict with microbial pathogens stands as a defining difficulty in the complex world of health and wellness. In this account, plant endophytic fungi create bioactive substances that may be helpful in the effort to safeguard human health [62]. The emergence of antimicrobial resistance poses a formidable threat to human health, demanding innovative solutions to combat bacterial, fungal, and viral pathogens. Traditional antibiotics are becoming less effective, which requires a change in the approach to microbial infections. In this context, there is a possibility that bioactive compounds from endophytic fungi can serve as new antimicrobial agents with significant potential [63]. These compounds, ranging from alkaloids to terpenoids, have the remarkable capacity to inhibit the growth and proliferation of a diverse spectrum of pathogens [64]. Their multifaceted mechanisms of action make them formidable contenders in the battle against microbial infections. Endophytic substances have antimicrobial properties that go beyond simple inhibition. They hold the potential to fortify the body’s defenses against pathogens [11]. By disrupting microbial cell membranes, inhibiting crucial enzymes, or interfering with their vital processes, these compounds actively combat microbial invaders, curbing their ability to cause infections [65]. *Aspergillus* sp. ASCLA was obtained from the leaf tissues of the medicinal plant *Callistemon subulatus*. From this, isoshamixanthone was extracted, displaying antimicrobial properties against pathogenic microorganisms [66]. Furthermore, a study was conducted on the isolation of *A. flavus* from *Ocimum basilicum*, revealing its effectiveness in inhibiting the growth of *Staphylococcus aureus*, *Bacillus cereus*, *B. subtilis*, *Escherichia coli*, *Salmonella typhimurium*, *Pseudomonas aeruginosa*, *Klebsiella pneumonia*, and *Candida albicans* at a concentration of 1000 µg/mL, showcasing antibacterial and antifungal attributes [15]. Another noteworthy group of endophytic ascomycetes is the *Penicillium* genus, frequently found within plant hosts. Recent investigations highlight the antibacterial and antifungal potentials exhibited by *Penicillium* against resilient microbes. An instance involves an endophytic *Penicillium* sp. isolated from the host plant *Stephania dielsiana*, which was tested against seven distinct pathogenic bacteria, demonstrating encouraging antimicrobial effectiveness [67]. A separate study documented the antimicrobial capability of *P. citrinum* derived from *Azadirachta indica*, manifesting activity against both human pathogenic bacteria and fungi [55].

These antimicrobial properties have implications for a full range of health and illness. These substances provide a comprehensive strategy for defending human health, dealing with both common infections and emerging and re-emerging pathogens. Furthermore, their potential for reducing biofilm formation, a major factor in the development of persistent infections, emphasizes their importance in contemporary healthcare strategies [68].

### 3.4. Anticancer Potential

The battle against cancer looms as a major obstacle in the complex world of health and medical development. The bioactive compounds derived from endophytic fungi within plants emerge as a novel avenue to combat this formidable disease [69]. Cancer remains one of the most complex and devastating diseases, challenging medical science to develop innovative strategies for prevention and treatment. As conventional therapies face limitations, the search for alternative solutions has intensified [70]. Hidden within the microcosm of endophytic fungi lies a reservoir of bioactive compounds that exhibit profound anticancer potential. These substances have shown the ability to inhibit the growth of cancer cells and trigger apoptosis (programmed cell death), ranging from the categories of alkaloids to polyphenols, and inhibit metastasis, which are the main characteristics of an effective anticancer agent [71]. The anticancer potential of endophytic compounds transcends mere cell inhibition. They engage intricate mechanisms that target cancer cells while sparing healthy ones. By modulating signaling pathways, interfering with vital cellular processes, and disrupting the microenvironment that supports cancer growth, these compounds offer a multi-pronged approach to cancer therapy [72]. In recent years, the prevailing genus among various other genera of fungal endophytes has been found to be *Aspergilli*. Studies have consistently reported the notable potential of Aspergilli in terms of their anticancer activity [55]. An illustrative example involves the endophytic strain *Aspergillus* TRL1, isolated from *Tabebuia rosea*, which has been harnessed to produce pulchranin, which is an effective anticancer compound. This compound demonstrated significant inhibitory effects against human tumor cells, including those of the liver (Hep-G2) and breast (MCF-7) [73]. Furthermore, the discovery of novel pyrano xanthones with anticancer properties emerged from *Aspergillus* ASCLA. These compounds exhibited anticancer efficacy against human cervix carcinoma [15]. The implications of the anticancer potential extend across the spectrum of cancer types, underscoring their relevance in reshaping the landscape of cancer treatment. From traditional malignancies to aggressive and drug-resistant cancers, the ability of these compounds to target diverse cellular vulnerabilities opens up new avenues for therapeutic innovation [74].

### 3.5. Immunomodulatory Effects

The proper functioning of the immune system is a crucial determinant of overall well-being. The bioactive compounds derived from endophytic fungi within plants emerge as intriguing agents capable of influencing the delicate balance of immune responses [75]. The immune system is a symphony of cells, signals, and responses, intricately choreographed to defend the body against threats and maintain equilibrium. A well-balanced immune response is vital for preventing infections, combating diseases, and preserving cellular health [76]. The study of bioactive compounds from endophytic fungi has opened a new chapter in the effort to use immunity for therapeutic purposes. These compounds, which range in nature from terpenes to glycosides, have the unique ability to regulate the immune system’s reactions. Whether it is enhancing immune defenses or damping down excessive responses, their actions are poised to revolutionize immune-related interventions [77]. The immunomodulatory effects of endophytic compounds extend beyond generalized immune stimulation. They involve the intricate regulation of immune responses by achieving a dynamic equilibrium between the activation and control of immune cells. This precise modulation carries the potential to mitigate autoimmune conditions, enhance the body’s ability to combat infections, and optimize the effectiveness of immune-mediated therapeutic approaches [78]. These compounds offer an avenue for tailored therapeutic interventions, from autoimmune disorders such as rheumatoid arthritis to enhancing vaccine responses. Moreover, their potential to counteract immune dysregulation associated with chronic diseases underscores their relevance in modern healthcare strategies [79].

## 4. Mechanisms of Action

Gaining insights into the sophisticated mechanisms underlying the health-promoting attributes of bioactive compounds sourced from plant endophytic fungi resembles the endeavor of unraveling the complexities of a symphony [80]. These health-promoting effects lie in a network of intricate molecular pathways. These pathways form the foundation upon which these compounds exert their transformative influence. These substances move through a web of pathways that intersect and converge to support health and well-being, from signaling cascades that control inflammation to complex biochemical reactions that support antioxidant defenses [2]. Bioactive substances do not just happen to have health-promoting properties. These interactions with specific cellular targets are precisely guided. For receptors, enzymes, and other molecules in cells, these substances have an astounding affinity. By attaching to these targets, they set off a chain of events that ripple through cellular processes, changing their course and guiding them towards outcomes that are beneficial for health. One of the most remarkable facets of these compounds is their ability to influence gene expression [81]. They are molecular architects capable of modulating the activity of genes, orchestrating a symphony of genetic responses that shape health outcomes. By upregulating protective genes, downregulating pro-inflammatory signals, and even affecting epigenetic modifications, these compounds have the power to influence health at a fundamental genetic level. The mechanisms underlying these health-promoting effects are not isolated; they intertwine and harmonize, culminating in a collective impact that goes beyond the sum of its parts [82]. The antioxidant activity interacts with anti-inflammatory pathways, the immunomodulatory effects intersect with cellular targets, and gene expression alterations ripple through multiple health dimensions. This integration of mechanisms underscores the complexity of these compounds’ actions and the potential they hold for transformative health outcomes [83]. As the journey through these mechanisms unfolds, the potential for precision health becomes evident. In the future, it is possible to use these substances to target particular health issues, adjusting interventions to unique genetic predispositions. Researchers are paving the path for novel therapeutics that will fully utilize the potential of endophytic chemicals for health promotion by elucidating the complexities of these mechanisms [84].

## 5. Potential Applications in Health and Medicine

### 5.1. Development of Pharmaceutical Drugs

In the complex world of modern medicine, scientists are actively searching for new and creative ways to find special substances in nature that can be used to develop better treatments for diseases [10]. The bioactive compounds from endophytic fungi hold the promise of becoming novel drug candidates, offering unique pathways for addressing a spectrum of health concerns. These compounds introduce innovative avenues for pharmaceutical development, from antimicrobial agents that combat resistant pathogens to anti-inflammatory drugs that modulate immune responses [85]. These candidates could stand as a vanguard against health challenges that have proven elusive to conventional treatments. One of the compelling aspects of bioactive compounds lies in their specificity. Unlike broad-spectrum drugs, these compounds have the potential to target specific cellular pathways, receptors, or molecules. This precision allows for the creation of targeted therapies that address the root causes of diseases without causing widespread collateral damage to healthy cells. This approach aligns with the evolving paradigm of personalized medicine [86]. The complexity of many diseases necessitates multifaceted treatment strategies. Bioactive compounds from endophytic fungi can play a pivotal role in such multimodal approaches. By harnessing their diverse health-promoting properties, pharmaceutical drugs can be designed to target multiple aspects of a disease simultaneously. This synergistic approach has the potential to enhance treatment outcomes while minimizing potential side effects [87].

Bioactive molecules offer a rich resource of evolutionarily selected potential medicinal agents. Scientists are able to decode the mechanisms of the chemicals, enhance their activity, and turn them into effective medications that tackle urgent health issues through methodical research and development. Challenges remain despite the amazing promise of bioactive molecules in medication development. Rigorous testing, optimization of dosages, and navigating the intricate interplay of compounds and the human body are crucial steps. The process demands a balance between innovation and meticulous assessment to ensure that the transformative potential of these compounds translates into safe and effective pharmaceutical solutions.

### 5.2. Exploration of Novel Pharmaceuticals from Microbial Origins: Utilizing Bioactive Compounds of Endophytic Fungi in Functional Foods

The pursuit of new pharmaceutical products derived from microbial sources traces its roots back to significant milestones such as the discovery of the anticancer drug “Taxol” from *Taxomyces andreanae* in the early 1990s and W. Flemming’s isolation of penicillin from *Penicillium notatum* in 1928 [10]. These pioneering breakthroughs in drug discovery involved compounds obtained from fungi. For example, Taxol was initially isolated from *Taxus brevifolia* and subsequently from *Taxus wallinchiana*, both of which host endophytic fungi, specifically *Taxomyces andreanae* and *Pestalotiopsis microspore*, respectively [88]. The emergence of these anticancer and antibiotic agents opened new avenues for uncovering biologically sourced drugs. Within this context, numerous bioactive compounds produced by endophytic fungi have demonstrated remarkable efficacy against antibiotic-resistant pathogens, positioning them as essential players in combating microbial infections [89]. However, the production and efficacy of these secondary metabolites are influenced by a range of factors, including sample collection timing, environmental conditions, habitat types (ranging from extreme environments such as saline habitats, high altitudes, rainforests, deserts, swamps, and marshes), plant host tissues (roots, leaves, and seeds), and the types of plants (angiosperms and gymnosperms) [90].

According to the literature, optimal conditions such as a healthy state of plant samples, are conducive to the selection of bioactive compounds. Furthermore, factors such as soil pH, temperature, humidity, light intensity, soil type, and microbiota contribute to the composition of these compounds [91]. Several instances from research articles highlight the isolation of potent antimicrobial compounds from endophytic fungi. For instance, the extraction of linoleic acid, R-glycerol monolinoleate, bisdethio-(bis-methyl-thio)-gliotoxin, fumiquinazoline-F, fumiquinazoline-D, and other compounds from *Aspergillus fumigatus* demonstrated significant antimicrobial effects [92]. Ethyl acetate extracts of endophytic Aspergilli have shown promising antibacterial and antifungal activity, accompanied by the production of antioxidant secondary metabolites such as alkaloids, terpenoids, and ρ-terphenyls. Moreover, endophytic fungi have unveiled a rich reservoir of bioactive compounds that extend beyond microbial resistance [93]. Notably, *Taxomyces andreanae*, isolated from the Pacific yew plant *Taxus brevifolia*, produces paclitaxel, or Taxol, which has gained prominence as an essential cancer treatment. Similarly, endophytes from various plants have been found to generate paclitaxel in different host species. Nonetheless, establishing a successful synthetic source of paclitaxel remains elusive [93]. Recent studies have also highlighted the versatile capability of endophytic fungi, such as *Alternaria* sp., in producing a range of active substances with various pharmacological activities, including cytotoxic, anti-trypanosomiasis, and anti-leishmaniasis effects. Additionally, bioactive compounds from endophytic fungi such as *Berkleasmium* sp., such as Diepoxin, Palmarumycin C11, Palmarumycin C12, and others, exhibit potent antifungal properties [93]. In light of these findings, the diverse array of bioactive compounds synthesized by endophytic fungi presents promising prospects for application in medical science, food, cosmetics, agriculture, and other industries. The classification of these secondary metabolites into categories such as alkaloids, terpenoids, steroids, polyketones, peptides, flavonoids, furandiones, quinols, perylene derivatives, and depsipeptides highlights their vast potential [93]. These compounds, borne from the intricate relationship between endophytic fungi and their host plants, signify an untapped reservoir of possibilities in the realm of functional foods and beyond. Through these explorations, the potential to revolutionize the treatment landscape and offer novel therapeutic options that prioritize both precision and patient well-being becomes increasingly evident [10].

## 6. Conclusions

In conclusion, the health-enhancing attributes of bioactive compounds derived from plant endophytic fungi shine as a beacon of promise for the future of healthcare and medicine within the intricate interplay of nature and science. These compounds offer a wide array of applications, ranging from their immunomodulatory capabilities to their antioxidant potency, and from the development of innovative treatments to the enhancement of daily diets. Their antioxidant properties act as formidable defenses against cellular deterioration, while their anti-inflammatory effects quell the flames of chronic inflammation. With their remarkable antimicrobial abilities, they target viruses with precision, and in the battle against cancer, they hold potential for cutting-edge therapeutic approaches. As they interact with molecular pathways, they influence gene expression, and their finesse in immunomodulation underscores their capacity to orchestrate immune responses.
